# Acquaintance, attitude, and perceived barriers regarding scientific research publications among clinical nurses: a cross-sectional study at tertiary care hospitals in western Rajasthan

**DOI:** 10.3389/frma.2024.1423963

**Published:** 2024-10-28

**Authors:** Nipin Kalal, N. sabari Vel, Saroj Chaudhary, Savita Meena, Sonam Meena, Sonu Bhichar, Spraha Singh

**Affiliations:** College of Nursing, AIIMS Jodhpur, Jodhpur, India

**Keywords:** scientific research, acquaintance, attitude, perceived barriers, clinical nurses

## Abstract

**Introduction:**

In today's era, conducting nursing research is crucial for the advancement of the nursing profession. Scientific publications in clinical research aim to improve patient care outcomes and foster a sense of importance for nurses within the healthcare team. However, clinical nurses often fall behind due to factors such as limited familiarity, attitudes toward research, and encountered barriers.

**Objectives:**

This study was conducted to assess the knowledge, attitude, and perceived barriers regarding scientific research publications among clinical nurses at tertiary care hospitals in western Rajasthan.

**Methodology:**

A cross-sectional descriptive study was conducted among the clinical nurses. The participants were assessed for their knowledge, attitude, and perceived barriers regarding scientific research publications through a self-administered questionnaire.

**Results:**

The study revealed that 92% of the participants lacked sufficient knowledge about scientific research publications and 78.3% experienced moderate perceived barriers. Pearson's correlation coefficient indicated a weak positive correlation (*r* = 0.143, *p* = 0.007) between knowledge and attitude and a significant negative correlation (*r* = −0.143, *p* = 0.012) between knowledge and perceived barriers. However, multiple linear regression analysis showed no significant relationship among the clinical nurses in terms of knowledge, attitude, and perceived barriers toward scientific research publications.

**Conclusion:**

This study on clinical nurses revealed that the majority had insufficient knowledge about scientific research, while over half had neutral attitudes toward research publications. In addition, a significant portion of the clinical nurses reported experiencing moderate perceived barriers.

## Introduction

Nursing research enhances patient care outcomes and fosters a perception of nurses as crucial and valuable members of the healthcare team (Jahan et al., 2015). Therefore, to empower and facilitate nurses, it is essential to provide administrative support and ensure proper nursing education for their active participation in the research process and the application of research findings in clinical practice (Al-Hussain et al., 2011). In the field of nursing, numerous factors influence academic research productivity. Alghanim and Alhamdi primarily distinguished between individual and institutional factors. According to them, individual factors included age, academic ranking, and experience, while institutional factors included availability of funds, departmental support, and institution size (Alghanim and Alhamali, 2011). Due to these factors, clinical nurses are struggling to keep up with conducting scientific research (Roberts and Turnbull, 2003).

Numerous studies have revealed that a limited number of nurses engage in research, which results in a slow progression and a lack of scientific awareness within the nursing profession (Tan et al., 2012). Nurses may make substantial contributions to patient care and improve health outcomes by enhancing their knowledge and applying it through research. Nurses who do not adhere to evidence-based practices often depend on established procedures and conventional nursing methods (Larsson and Thorslund, 2006). Despite the availability of research-based knowledge and interventions, some nurses still adhere to outdated methods in healthcare (Grol and Grimshaw, 2003).

Nursing care plays one of the crucial roles in the healthcare delivery system. Patient satisfaction with nursing care is determined by the quality of the healthcare professionals. This quality is achieved through scientific research knowledge and evidence-based practices (Buchanan et al., 2015). The World Health Organization reported a study on the diabetes knowledge of nurses in different countries. The report highlighted that knowledge forms the basis of professional practice and that deficits in diabetes care knowledge represent a major risk of unsafe practices. Proper continuing education and scientific research will help advance the nursing profession (Alotaibi et al., 2016).

Currently, the Medical Council of India (MCI) has mandated that faculty seeking promotion to higher positions must reassess the publication criteria established jointly by the MCI and the National Medical Commission (NMC) Bill (Joseph et al., 2023). According to a statement from the Ministry of Human Resource Development in 2022, the National Institutional Ranking Framework provided an overview of each institution's status, compiling data across five key categories: Teaching Learning and Resources, Research and Professional Practice (RPP), Graduation Outcomes, Outreach and Inclusivity, and Perception. Our institute has been ranked 16th based on this categorization. To address short comings and enhance our overall ranking, a study has been initiated, focusing particularly on research and publications, where our institute currently holds a cumulative score of 57.47/100 (Dandannavar et al., 2020).

Currently, many colleges are seeking national accreditation, highlighting the need for research productivity among faculty members. However, there is limited research on nursing faculty members' attitudes toward publishing scientific research and the challenges they face. The existing literature in India primarily concentrates on bedside nurses, medical personnel, or a combination of faculty members, thereby falling short of offering a thorough insight into the stance of nurses, specifically, regarding scientific research publications (Alotaibi et al., 2016). From the above-mentioned article, it is clear that scientific research publications are essential for enhancing the knowledge of nurses. We also anticipate that research outcomes may improve if any restraints are acknowledged and corrective actions are required to address them. It is also understood that very few studies have been conducted among clinical nurses. Therefore, this inference leads us to conclude that this is a novel study and that there is a pressing need to carry out this study in tertiary care centers.

## Methodology

### Study design

A hospital-based cross-sectional study was conducted among clinical nurses (*n* = 350) from March to April 2023.

### Study setting and study participants

The criteria for selecting the sample included registered clinical nurses with atleast 1 year of clinical experience and nurses who are working in the in-patient department (IPD) and out-patient department (OPD). Deputy nursing superintendents, nursing superintendents, and chief nursing officers were excluded from the study.

The sample size was determined using the formula for a single population proportion, considering a margin of error of 5%, a confidence level of 95%, and an estimated moderate knowledge rate of 80.2% (Khan et al., 2009). Based on these parameters, we determined that a minimum sample size of 245 was necessary. Nevertheless, we intentionally opted to select 350 clinical nurses employed at AIIMS Jodhpur for our study.

### Steps for the selection of the study participants

1. The researcher selected study participants who were working in the IPD and OPD wards at AIIMS Hospital, Jodhpur.
2. The participants were selected based on the screening done by the researcher, and those who met the inclusion criteria were assigned one by one following a sequence. A total of 350 participants were selected for the main study, and a total of five participants were excluded.

### Data collection tool and technique

A self-administered questionnaire was constructed using a 3-point Likert scale to assess the participants' level of knowledge. It included questions related to the knowledge and experience of the participants regarding research work. The scale consisted of 10 items. Each accurate answer received a score of 1, while an incorrect response received a score of 0.

The total number of possible correct responses was 10. The scores were interpreted as follows: below 50% indicated inadequate acquaintance, 51%−75% indicated moderate adequate acquaintance, and 76%−100% indicated adequate acquaintance.

The attitude toward scientific research publications was assessed using a 5-point Likert scale of 10 items. The scores were interpreted as follows: below 50% indicated a negative attitude, 51%−75% indicated a neutral attitude, and 76%−100% indicated a positive attitude. The perceived barriers among the participants were assessed using a 3-point Likert scale. It has two domains (institutional and individual barriers) comprising 10 items. The scores were interpreted as follows: scores between 0–7 indicated low perceived barriers, scores between 8 and 14 indicated moderate perceived barriers, and scores between 15 and 20 indicated high perceived barriers.

The validity of the tools was established based on expert opinion and the scale-content validity index (S-CVI) with a score of 0.88, indicating that the tool is acceptable and valid. Cronbach's alpha was used to determine the reliability of the components. The tool was confirmed to be dependable, and the reliability of the components is as follows: acquaintance −0.88, attitude −0.91, and barrier −0.86.

### Statistical analysis

The data were coded and entered into an Excel spreadsheet, and then, they were analyzed using SPSS software version 16 (IBM Inc., Armonk, New York, USA). Descriptive analysis was performed to examine the participant demographics in terms of frequency and percentage. Multiple linear regression analysis was performed to investigate the correlation between acquaintance, attitude, and perceived barriers. In addition, a chi-squared test was applied to assess the associations between the categorical variables and acquaintance, attitude, and perceived barriers, with a significance level set at a *p*-value of < 0.05 to indicate statistical significance.

### Ethical consideration

The study was approved by the Institutional Ethics Committee of AIIMS, Jodhpur, with the reference number AIIMS/IEC/2023/4347. The study participants received an explanation of the data collection process, and their informed consent was obtained before participation. Strict confidentiality measures were upheld throughout the study.

## Results

Table 1 shows the sociodemographic variables. More than half of the clinical nurses, 52.9%, belonged to the age group of 20–30 years. In terms of gender distribution, nearly two-thirds, 65.7%, were men and nearly one-third, 34.3%, were women. Regarding the marital status of the participants, 77% were married. In regards to educational qualification, 78.9% of the clinical nurses had a degree in B.Sc. Nursing. Furthermore, 40% of the participants had a clinical experience of 3–6 years. The majority of the participants, 70.9%, were nursing officers. Additionally, 42.6% of the participants reported their area of work as IPD. Nearly half of the respondents, 47.1%, mentioned that they had completed their most recent professional education at state government institutes. Among the respondents, 86.3% had not published any research papers, while only 13.7% had prior publications.

**Table 1:** Sociodemographic variables of the participants.

**Sociodemographic variables**	**Frequency (percentage) *N* = 350**
**Age (in years)**	
20–30	185 (52.9%)
31–40	154 (44%)
41–50	11 (3.1%)
**Sex**	
Male	230 (65.7%)
Female	120 (34.3%)
**Marital status**	
Single	77 (22%)
Married	272 (77%)
Widow/widower	1 (0.3%)
**Education qualification**	
M.Sc. Nursing and above	16 (4.6%)
B.Sc. Nursing	276 (78.9%)
Post Basic. B.Sc. Nursing	40 (11.4%)
GNM	18 (5.1%)
**Clinical experiences**	
1–3 years	70 (20%)
3–6 years	140 (40%)
6–10 years	96 (27.4%)
More than 10 years	44 (12.6%)
**Designation**	
Nursing officer	248(70.9%)
Senior nursing officers	85 (24.3%)
Assistant nursing superintendent	17(4.9%)
**Area of posting/working**	
OPD	9 (2.6%)
IPD	149 (42.6%)
OT and ICUs	110 (31.4%)
Emergency complex	82 (23.4%)
**Last professional education completed from**	
Central government	83 (23.7%)
State government	165 (47.1%)
Private	102 (29.1%)
**Have you published any research papers?**	
Yes	48 (13.7%)
No	302 (86.3%)

Table 2 shows that only 1.7% of the participants had adequate acquaintance with scientific research publications, while the majority of them, 92%, had inadequate acquaintance. The level of knowledge showed a mean and standard deviation of 1.10 ± 0.350. Regarding attitude, more than half, 67.1%, of the participants had a neutral attitude toward research publications, followed by 32% who had a positive attitude and 0.9% who had a negative attitude. The level of attitude showed a mean and standard deviation of 2.31 ± 0.482. Most of the participants, 78.3%, experienced moderate perceived barriers, while 11.4% experienced low perceived barriers and 10.3% experienced high perceived barriers. The level of perceived barriers showed a mean and standard deviation of 1.99 ± 0.467.

**Table 2:** Participant's acquaintance, attitude, and perceived barriers regarding scientific research publications.

**Parameter**	***N*** = **350**	
	**Frequency (percentage)**	**Mean (SD)**
**Acquaintance score**	
Inadequate acquaintance	322 (92%)	1.10 (0.350)
Moderately adequate acquaintance	22 (6.3%)	
Adequate acquaintance	6 (1.7%)	
**Attitude score**	
Negative attitude	3 (0.9%)	2.31 (0.482)
Neutral attitude	235 (67.1%)	
Positive attitude	112 (32%)	
**Perceived barrier score**	
Low perceived barriers	40 (11.4%)	1.99 (0.467)
Moderate perceived barriers	247 (78.3%)	
High perceived barriers	36 (10.3%)	

The correlation between acquaintance, attitude, and perceived barriers was assessed using Pearson's correlation coefficient. A significant weak positive correlation was found between acquaintance and attitude (*r* = 0.143, *p* = 0.007) and between perceived barrier and attitude (*r* = 0.003, *p* = 0.953). A significant negative correlation was found between acquaintance and perceived barrier (*r* = −0.143, *p* = 0.012; Table 3).

**Table 3:** Correlation between acquaintance, attitude, and perceived barriers regarding scientific research publications among the clinical nurses.

**Variables**	**Correlation coefficient**	***p*-value**
Acquaintance and attitude	0.143	0.007
Acquaintance and perceived barriers	−0.134	0.012
Perceived barriers and attitude	0.003	0.953

Table 4 shows the results of the multiple linear regression analysis examining the relationship between acquaintance, attitudes, and perceived barriers. There was no significant relationship between acquaintance, attitude, and perceived barriers regarding scientific research publications among the clinical nurses.

**Table 4:** Multiple linear regression analysis of the relationship between acquaintance, attitude, and perceived barriers toward scientific research publications among the clinical nurses.

**Variables**	**Perceived barriers**	***p*-value**
	**Regression coefficient**	
Acquaintance	−2.548 (−0.324 to −0.042)	0.011
Attitude	0.423 (−0.080 to 0.124)	0.673

There was an association between knowledge, attitude, and perceived barriers and the selected demographic variables regarding scientific research publications among the clinical nurse. The level of knowledge was associated with the sociodemographic variable educational qualification (*p* = 0.011). Attitude showed a significant association with marital status (*p* = 0.024), designation (*p* = 0.019), area of work (*p* = 0.010), and previous publication of research papers (*p* = 0.006). The level of perceived barriers had a significant association with gender (*p* = 0.001) and marital status (*p* = 0.003; Table 5).

**Table 5:** Association between knowledge, attitude, and perceived barriers and selected demographic variables.

**Demographic variables**	**Acquaintance**		**Attitude**		**Perceived barriers** ***N*** = **350**	
	**Mean (SD)**	***p*** **-value**	**Mean (SD)**	***p*** **-value**	**Mean (SD)**	***p*** **-value**
**Age (in years)**						
20–30	1.08 (0.328)	0.803	2.32 (0.490)	0.539	1.97 (0.453)	0.300
31–40	1.08 (0.328)		2.32 (0.481)		2.01 (0.498)	
41–50	1.09 (0.302)		2.09 (0.302)		2.00 (0.000)	
**Sex**						
Male	1.07 (0.287)	0.118	2.29 (0.465)	0.178	2.06 (0.449)	0.001^*^
Female	1.15 (0.442)		2.35 (0.513)		1.86 (0.473)	
**Marital status**						
Single	1.08 (0.315)	0.982	2.42(0.547)	0.024^*^	1.96 (0.572)	0.003^*^
Married	1.10 (0.360)		2.28 (0.459)		1.99 (0.429)	
Widow/widower	1.00 (0)		2.00 (0)		3.00 (0)	
**Education qualification**						
M.Sc. Nursing and above	1.31 (0.704)	0.011^*^	2.56 (0.512)	0.165	2.00 (0.365)	0.934
B.Sc. Nursing	1.09 (0.328)		2.32 (0.488)		1.99 (0.475)	
Post Basic. B.Sc. Nursing	1.03 (0.158)		2.18 (0.385)		2.00 (0.453)	
GNM	1.11 (0.471)		2.33 (0.485)		1.89 (0.471)	
**Clinical experiences**						
1–3 years	1.09 (0.371)	0.249	2.36 (0.512)	0.674	1.90 (0.542)	0.140
3–6 years	1.13 (0.395)		2.30 (0.490)		1.96 (0.455)	
6–10 years	1.05 (0.266)		2.32 (0.470)		2.04 (0.433)	
More than 10 years	1.11 (0.321)		2.25 (0.438)		2.09 (0.421)	
**Designation**						
Nursing officer	1.08 (0.332)	0.665	2.29 (0.465)	0.019^*^	2.02 (0.458)	0.185
Senior nursing officers	1.13 (0.402)		2.29 (0.508)		1.92 (0.493)	
Assistant nursing superintendent	1.12 (0.332)		2.65 (0.493)		1.82 (0.393)	
**Area of posting/working**						
OPD	1.00 (0.000)	0.613	1.06 (0.241)	0.010^*^	2.11 (0.601)	0.070
IPD	1.13 (0.414)		2.21 (0.459)		1.90 (0.476)	
OT and ICUs	1.08 (0.335)		2.45 (0.499)		2.04 (0.427)	
Emergency complex	1.06 (0.241)		2.32 (0.468)		2.07 (0.466)	
**Last professional education completed from**						
Central government	1.10 (0.335)	0.515	2.27 (0.496)	0.328	1.98 (0.517)	0.657
State government	1.09 (0.363)		2.32 (0.466)		2.01 (0.442)	
Private	1.11 (0.342)		2.34 (0.497)		1.96 (0.465)	
**Have you published any research papers?**						
Yes	1.08 (0.321)	0.155	2.30 (0.467)	0.006^*^	2.00 (0.450)	0.084
No	1.19 (0.491)		2.37 (0.570)		1.90 (0.555)	

^*^Significant at a p-value of < 0.05.

## Discussion

Evidenced-based practices provide the best possible treatment and care for patients; however, recent studies have revealed that clinical nurses are unable to participate in scientific research publications. The current study aimed to explore the reasons for fewer or poor scientific research publications such as the level of knowledge, the attitude, and the level of perceived barriers among clinical nurses. In this study, we found that 92% of the participants had inadequate knowledge regarding scientific research publication, while 6.3% had moderately adequate knowledge and 1.2% had adequate knowledge, with a mean score of 1.10 ± 0.350 and a maximum of 10. This finding is supported a previous study conducted among 295 undergraduate medical and dental students (Khan et al., 2009).

Among the students surveyed, 56.9% exhibited a moderate level of knowledge, while 39.1% demonstrated a poor level of knowledge. Furthermore, it is noted that the attitude toward health research serves as a significant predictor of both evidence-based practices and the utilization of healthcare research (Kyaw Soe et al., 2018). This study showed that 67.1% of the clinical nurses had a neutral attitude toward research publications. This finding is supported by another study, in which 83.3% of students had a neutral attitude. In addition, the current study showed that 32% of the participants had a positive attitude. This finding is supported by another study, in which 11.3% of participants had a good attitude. In the current study, the level of attitude showed a score range of 17–50 and a mean of 2.31 ± 0.482. Our study showed that 78.3% experienced moderate perceived barriers, while 11.4% experienced low perceived barriers and 10.3% experienced high perceived barriers, with a mean score of 1.99 ± 0.467 and a maximum of 20. Previous studies have shown that the most commonly cited barriers are a lack of time (79.9%), followed by a lack of knowledge and skills (72.1%) and a lack of funding (72.0%). Similar to other studies (Khan et al., 2009), a considerable portion of our participants demonstrated varying degrees of confidence in carrying out research tasks, including formulating clinical inquiries and conducting literature searches and evaluations. However, a significant number of them indicated a lack of confidence in accessing clinical guidance from instructors and implementing evidence-based methodologies. Health professionals who perceive themselves as capable of conducting research activities are more inclined to engage in nursing research (Brown et al., 2010). In our study, the level of knowledge was found to be associated with the sociodemographic variable educational qualification; however, the level of knowledge was not significantly associated with any other sociodemographic variables, such as designation or years of clinical experience. Furthermore, our study also showed that attitude had a significant association with the sociodemographic variables, which included marital status, designation, area of work, and previous publication of research papers. However, attitude was not significantly associated with any other sociodemographic variables. This is comparable to the research conducted among licensed nurses by Bonner and Sando (2018). Our findings suggest that comprehensive training in research principles, coupled with compulsory involvement in research activities, can lead to significant improvements in content knowledge and foster positive attitudes toward future research endeavors. However, despite the emphasis placed on promoting scientific research, the existence of barriers creates a disparity between theory and practice. These barriers to participating in scientific research can be categorized as individual hurdles, such as a lack of personal interest, self-confidence, and time constraints due to excessive workload and financial burdens. In addition, institutional barriers, including insufficient digital resources, funding and sponsorship shortages, and a lack of colleague motivation, mentorship, and awareness regarding submission processes, as well as inadequate training in research methodology, contribute to these challenges. In this study, the clinical nurses frequently cited lack of interest, insufficient resources, and time constraints as the primary obstacles they faced. Furthermore, the clinical nurses highlighted that limited accessibility to pertinent nursing and other electronic databases hinders their ability to identify knowledge gaps and initiate their research endeavors (Khan et al., 2009). Previous studies have indicated that the attitude toward involvement in research and its utilization play pivotal roles in evidence-based medicine and that negative attitudes may impede the implementation of scientific research. It is widely acknowledged that a supportive and positive environment can encourage successful researchers and significantly influence research output, including publications (Hickson, 2018).

## Limitations

The findings of our study should be appraised considering a few limitations. First, the study included only clinical nurses working in tertiary care centers. Second, considering the cross-sectional design of the study, the chances of subjectivity and responses might not represent the actual behavior of nurses. Third, our study lacked intervention implementation and involved one-time data collection, thereby restricting the generalizability of the study findings. Hence, future research endeavors should incorporate larger sample sizes and qualitative methodologies, such as focus group discussions, to attain a more comprehensive understanding of the issues. A comparable study could be conducted among college students and various healthcare professionals.

## Conclusion

There is an increased need for scientific research publications among nurses to improve healthcare practice and advance their field. However, either due to a lack of interest or a lack of a supportive environment, there is a dearth of publications by nurses. The majority of studies conducted on this subject focused on medical staff or faculty members. There is a lack of data from clinical nurses in India. This study concluded that the majority of the nurses have a neutral attitude and inadequate knowledge regarding scientific research publications. Evidence-based nursing practice must take a more prominent role in improving the quality of patient care. Therefore, institutions need to develop guidelines and protocols regarding scientific research publications through seminars, conferences, and other media. The nursing curriculum should include guidelines for writing scientific research publications and addressing plagiarism.

## Data Availability

The original contributions presented in the study are included in the article/supplementary material, further inquiries can be directed to the corresponding author.
